# Lampreys, the jawless vertebrates, contain three *Pax6* genes with distinct expression in eye, brain and pancreas

**DOI:** 10.1038/s41598-019-56085-8

**Published:** 2019-12-20

**Authors:** Vydianathan Ravi, Shipra Bhatia, Prashant Shingate, Boon-Hui Tay, Byrappa Venkatesh, Dirk A. Kleinjan

**Affiliations:** 1 Institute of Molecular and Cell Biology, Agency for Science Technology and Research (A*STAR), Biopolis, Singapore, Singapore; 2 MRC Human Genetics Unit, MRC Institute of Genetics and Molecular Medicine, University of Edinburgh, Edinburgh, United Kingdom; 3 Department of Paediatrics, Yong Loo Lin School of Medicine, National University of Singapore, Singapore, Singapore; 4 Present Address: Centre for Mammalian Synthetic Biology, University of Edinburgh, Roger Land building, Kings buildings, Edinburgh, EH9 3FF UK

**Keywords:** Genetics, Phylogenetics

## Abstract

The transcription factor *Pax6* is crucial for the development of the central nervous system, eye, olfactory system and pancreas, and is implicated in human disease. While a single *Pax6* gene exists in human and chicken, *Pax6* occurs as a gene family in other vertebrates, with two members in elephant shark, *Xenopus tropicalis* and Anolis lizard and three members in teleost fish such as stickleback and medaka. However, the complement of *Pax6* genes in jawless vertebrates (cyclostomes), the sister group of jawed vertebrates (gnathostomes), is unknown. Using a combination of BAC sequencing and genome analysis, we discovered three *Pax6* genes in lampreys. Unlike the paired-less *Pax6* present in some gnathostomes, all three lamprey *Pax6* have a highly conserved full-length paired domain. All three *Pax6* genes are expressed in the eye and brain, with variable expression in other tissues. Notably, lamprey *Pax6α* transcripts are found in the pancreas, a vertebrate-specific organ, indicating the involvement of *Pax6* in development of the pancreas in the vertebrate ancestor. Multi-species sequence comparisons revealed only a single conserved non-coding element, in the lamprey *Pax6β* locus, with similarity to the *PAX6* neuroretina enhancer. Using a transgenic zebrafish enhancer assay we demonstrate functional conservation of this element over 500 million years of vertebrate evolution.

## Introduction

*Pax6* is an evolutionarily conserved, pleiotropic transcription factor with key roles during embryonic and postnatal development as well as in adult tissue maintenance. In both vertebrates and invertebrates *Pax6* acts as a master regulator controlling multiple genetic networks that drive differentiation and cell type specification. In vertebrates *Pax6* is essential for proper development of the central nervous system (CNS), the eye, and the olfactory system^1–4^. It is also crucial for the development of the pancreas and subsequent insulin production from its endocrine secretory cells^5–8^. A correct dosage of *Pax6* is essential for proper eye development. Haploinsufficiency leads to the congenital eye malformation aniridia in humans and the *small eye* mutation in mice, whereas loss of both alleles causes a complete lack of eye development and results in congenital lethality^9^. *Pax6* is expressed in all tissues of the embryonic eye. Spatio-temporally restricted ablation of the gene in conditional mouse mutants has revealed tissue-specific requirements in lens and retina development^10–12^. Interestingly, while human, mouse and chicken possess a single *Pax6* gene (*Pax6*.*1*), other gnathostomes such as elephant shark (*Callorhinchus milii*), *Xenopus tropicalis* and Anolis lizard contain two *Pax6 genes*^13^, known as *Pax6*.*1 and Pax6*.*2* (the latter is also referred to as *Pax10*^14^). The two genes in the latter taxa have been attributed to two rounds of whole-genome duplication (WGD), commonly referred to as 2 R, that occurred at the base of vertebrates^15,16^, followed by secondary loss of two paralogs^13^. One more gene was subsequently lost in the lineage leading to mammals and birds. A third *Pax6* paralog is present in many teleost fish such as fugu (*Takifugu rubripes*), medaka (*Oryzias latipes*) and stickleback (*Gasterosteus aculeatus*), where, as a result of a further teleost-specific WGD and subsequent separate gene losses, a variable complement of *Pax6* paralogs is present in contemporary lineages^13,14^. However, the number of *Pax6* genes in cyclostomes, the jawless vertebrates, is currently unknown.

Inter-species sequence comparisons of the *Pax6* genomic region have aided in the identification of a large number of enhancer elements, revealing a highly complex *cis*-regulatory landscape surrounding the gene. The importance of these *cis*-regulatory elements (CREs) is exemplified by a subset of aniridia patients in whom, even though the *PAX6* sequence itself remains intact, regulation of the gene is disrupted due to nearby chromosomal breaks^17^. A large number of these CREs can be traced back to the common ancestor of gnathostomes, as shown by their presence in the elephant shark *Callorhinchus milii*^13,18^. In this study, we traced back further in evolution and investigated the presence of *Pax6* genes and their regulatory landscapes in cyclostomes.

Cyclostomes are the sister group of the gnathostomes. They split from the jawed vertebrates very early during vertebrate evolution, estimated to be around 500 million years ago. Cyclostomes are a monophyletic group^19^ whose only extant members are the lampreys and hagfishes. As these species represent the earliest branching lineage of the vertebrates, they form a key resource for understanding the molecular events that occurred during the early evolution of vertebrates. Evaluation of the gene content in lampreys and hagfishes might therefore shed light on the pattern of gene and genome duplications in early vertebrates. However, the interpretation of the findings might be complicated due to recent reports suggesting a third round of WGD in lampreys^20^, although evidence from another study instead points towards a scenario of segmental duplications of several, but not all, genomic loci^21,22^.

In all analysed gnathostome species *Pax6* exhibits a highly tissue-specific expression pattern. Control of *Pax6* gene expression in lamprey is of interest from an eye evolution perspective as the eyes of adult lampreys are already similar to jawed-vertebrate eyes, possessing a lens, iris and three-layered retina. On the other hand, the hagfish eye appears more basic and lacks a proper lens and cornea, and has a simpler retina. This is outwardly similar to the simpler eyes found in the larval stage of lampreys, before they dramatically metamorphose into the adult form^23^. As *Pax6* is a master control gene for eye development, with its strict spatio-temporal expression pattern controlled by several highly conserved CREs, we were curious to investigate the evolutionary origin of eye-specific enhancers. *Pax6* also plays a key role in the development and maintenance of the endocrine pancreas^5,7,8^, a vertebrate-specific organ which exists in a simple form in cyclostomes where it is usually referred to as the islet organ^24^. Whereas in hagfish the islet organ is represented by scattered follicles, lampreys have a discrete islet organ associated with the gut. We were therefore interested to investigate whether *Pax6* expression is associated with pancreas development in lampreys.

We had previously generated a whole-genome sequence for the Japanese lamprey (*Lethenteron japonicum aka Lethenteron camtschaticum*)^20^. Using a combination of whole-genome sequence analysis and sequencing of BAC clones we identified three *Pax6* paralogs in the Japanese lamprey genome and designated them as *Pax6α*, *Pax6β* and *Pax6γ*. Analysis of the recently published germline genome assembly of the sea lamprey (*Petromyzon marinus*) showed that the three *Pax6* genes are also present in this lamprey^22^. A search for ancient conserved noncoding elements (aCNEs) identified a single element in the Japanese lamprey *Pax6β* locus. In contrast, no CNEs are detected in sequence comparisons between vertebrate and Amphioxus *Pax6* loci. Using a transgenic zebrafish reporter assay, we show that the conserved lamprey element is capable of driving specific expression in the neuroretina, revealing a remarkable functional conservation dating back to the origin of vertebrates.

## Results

### *Pax6* genes in the Japanese lamprey genome

**Figure 1:**
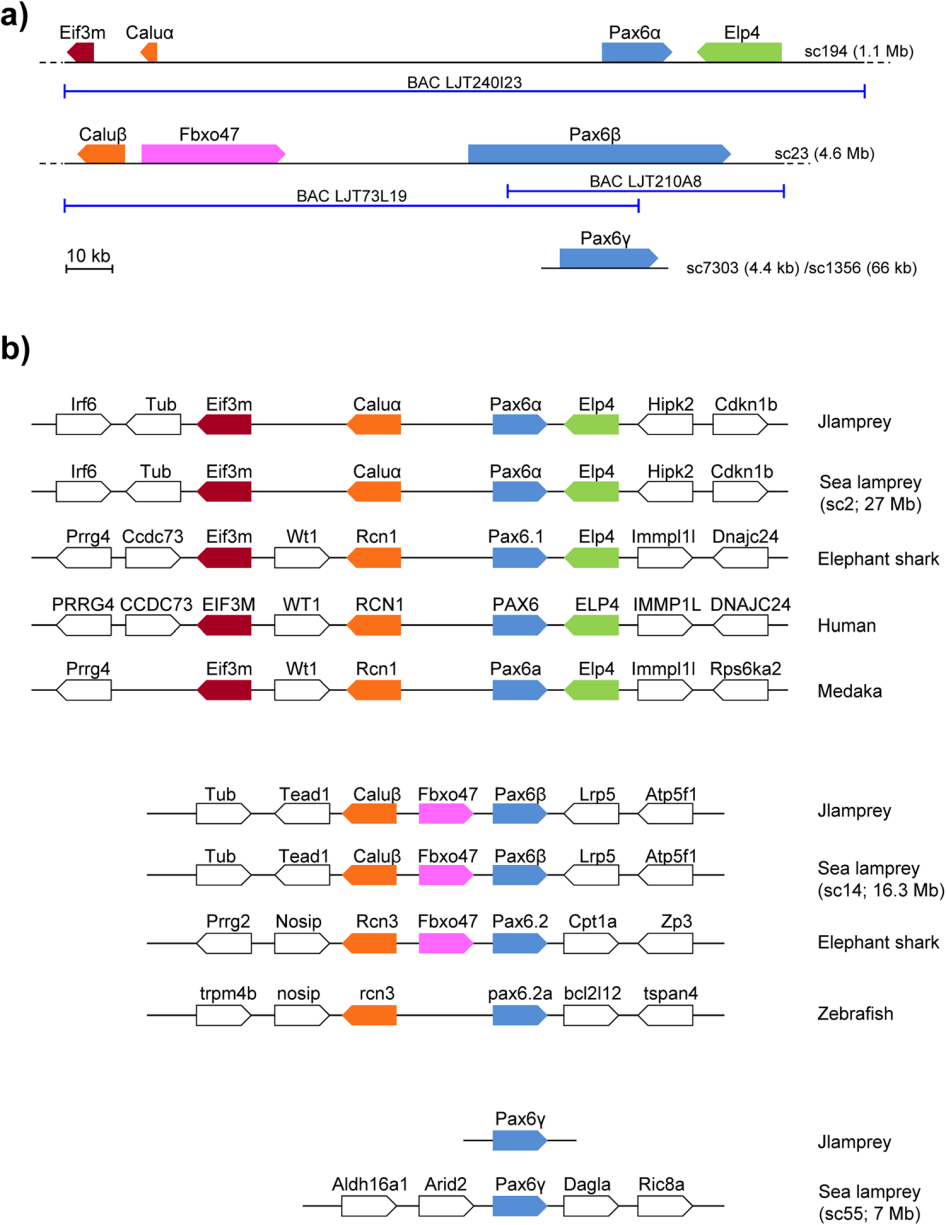
The lamprey genome contains three *Pax6* genes. (**a**) The three *Pax6* loci in the Japanese lamprey, *L*. *japonicum*. Lamprey *Pax6* genes are named *Pax6α*, *Pax6β* and *Pax6γ*. The BAC clones sequenced are shown below. *LjPax6γ* resides on a short (66 kb) scaffold containing no other genes. (**b**) Gene synteny comparison of the three Japanese lamprey (Jlamprey) and sea lamprey *Pax6* loci with *Pax6* loci from selected gnathostomes.

In order to identify *Pax6* genes in Japanese lamprey we searched its germline genome assembly^20^ using known *Pax6* protein sequences as TBLASTN queries. These searches identified three distinct *Pax6* gene fragments, present on scaffold 194 (1,091,192 bp), scaffold 23 (4,607,062 bp), and a third one distributed across multiple smaller scaffolds (scaffolds 7303, 22485, 72958, 1356, 12381 and 20282) (Fig. 1a). To obtain contiguous sequence for each of these loci, we used the identified scaffolds to design probes for screening of Japanese lamprey BAC libraries. We identified several BACs, of which three were sequenced completely. One of these (LJT240I23) covers the first *Pax6* locus (scaffold 194) whereas the remaining two (LJT73L19 and LJT210A8) are overlapping BACs covering the second *Pax6* locus (scaffold 23). We were unable to identify a BAC clone for the third *Pax6* locus. To obtain contiguous sequence for the third *Pax6* locus we used a combination of RT-PCR and RACE to identify and orient scaffolds belonging to the same *Pax6* gene and used genomic PCR to fill the intervening gaps. Synteny and sequence analysis confirmed that these three genes are distinct *Pax6* genes showing some level of conserved synteny with human and elephant shark *Pax6* gene loci (Fig. 1b). Since phylogenetic analysis was unable to assign clear orthology of the three lamprey genes to specific *Pax6* family members in gnathostomes (see Phylogenetic analysis section), we named the three Japanese lamprey *Pax6* genes as *Pax6α*, *Pax6β* and *Pax6γ*. Lamprey *Pax6β* had been sequenced and characterized in a previous study^25,26^.

**Figure 2:**
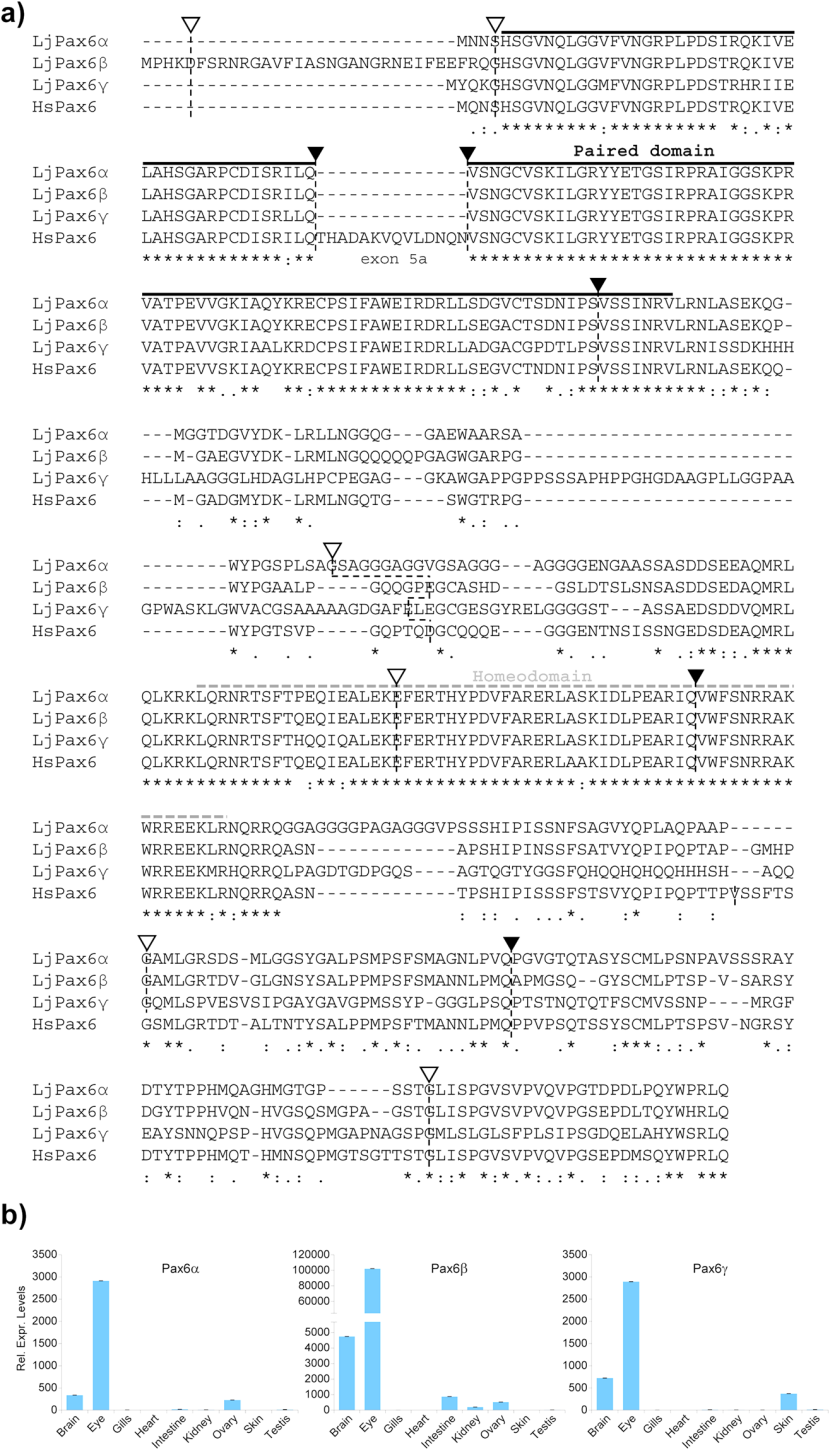
Protein sequences and expression patterns of the Japanese lamprey *Pax6* genes. (**a**) Comparison of the amino acid sequences of Lj*Pax6*α, Lj*Pax6*β and Lj*Pax6*γ with human (Hs) *PAX6*. All three *LjPax6* genes encode a highly conserved paired domain (solid black line), in contrast to known *Pax6*.*2* (also referred to as *Pax10*^14^) genes which lack the sequences coding for this domain, as well as a highly conserved homeodomain (dotted grey line) and C-terminal transactivation domain. No evidence was found for the presence of the alternative exon 5a in the lamprey genes. The positions of the exon boundaries (black arrow head, phase 0 intron; open arrow head, phase 1 intron) are conserved between the human and lamprey genes. (**b**) qRT-PCR analysis using a panel of adult lamprey tissues showing the tissue-specific expression pattern of the *LjPax6* genes. All three genes are highly expressed in the eye and brain, with lower and variable expression in other tissues.

*Pax6* is a transcription factor containing two distinct DNA-binding domains: the 128 amino acid long paired domain located at the N-terminal and the more centrally located homeo-domain. These are connected via a linker region while the C-terminus harbours a PST-rich transactivation domain^3^. All three lamprey *Pax6* protein sequences showed very high sequence similarity with each other as well as with the human *PAX6* over the paired- and homeo-domains (Fig. 2a), with lower similarity in the linker region and higher similarity again in the PST-domain. Alignment of the lamprey *Pax6* protein sequences revealed 79.7% identity between *Pax6*α and *Pax6*β, 58.3% identity between *Pax6*α and *Pax6*γ and 59.4% between *Pax6*β and *Pax6*γ.

### Gene structure of the Japanese lamprey *Pax6* genes

All three lamprey *Pax6* genes contain both the paired- and homeo-domains. This contrasts with gnathostome species that carry multiple *Pax6* genes in their genome, in which the *Pax6*.*2* gene lacks the N-terminal paired domain^13^. The intron-exon structure of the three genes is also fully conserved between the lamprey genes and the canonical human *PAX6* gene (Fig. 2a; Fig. S1). Both lamprey *Pax6*α and *Pax6*γ have only a few amino acids in their first coding exon before the start of the paired domain. This is equivalent to mammalian *Pax6* where the first coding exon (exon 4) encodes just three/four amino acids with the paired domain being encoded by exons 5, 6 and part of 7 (Fig. 2a).

### Absence of exon 5a in Japanese lamprey *Pax6* genes

Two major isoforms of the full-length *Pax6* exist in tetrapods, teleost fishes and elephant shark. These isoforms, called *Pax6* and *Pax6*(5a), differ by a stretch of 12 to 14 amino acids which are present in the latter isoform as an insertion in the PAI subdomain within the paired domain and change its binding site recognition characteristics^27,28^. A study searching EST and other databases for the *Pax6*(5a) isoform had previously found this isoform only in gnathostome species^29^. To check for the potential presence of this alternative exon in the Japanese lamprey *Pax6* genes we closely inspected the genomic sequence between exons 5 and 6 in the three *Pax6* genes. However, we were unable to find any sequence homologous to exon 5a in any of the three *Pax6* genes (Fig. 2a). RT-PCR products from the three genes also lack the bases coding for this exon. It is therefore most likely that exon 5a is an innovation that is specific to the gnathostomes.

### *Pax6* genes in the sea lamprey genome

Following the recent completion of the germline genome assembly of the sea lamprey^22^, we also searched its genome sequence for *Pax6* genes by TBLASTN using Japanese lamprey and other representative *Pax6* protein sequences as query. These two lamprey species are estimated to have diverged about 10 to 40 million years ago^30^. We identified three *Pax6* genes in the sea lamprey that are homologous to the *Pax6* genes of Japanese lamprey (Fig. 1b). They are present on scaffold_2: 14371676-14388243 (*Pax6α*), scaffold_14: 14097046-14127107 (*Pax6β*) and scaffold_55: 4629581-4632980 (*Pax6γ*). Some exons of *Pax6γ* are found on a short scaffold (scaffold_699, 41.7 kb). Since these genes are either incomplete or contain frame shifts and other errors, we could not predict reliable full-length protein sequences for them.

### Synteny relationships

Next we compared the synteny of genes at the three Japanese lamprey *Pax6* loci with those from sea lamprey and representative gnathostomes (Fig. 1b). Lamprey *Pax6α* is flanked by *Eif3m* and *Caluα* at the 5′ end and *Elp4* at the 3′ end, similar to the gnathostome *Pax6*.*1* (e.g. human *PAX6*) synteny region. The lamprey *Pax6*β locus contains *Caluβ* and *Fbxo47* genes. Linkage with *Calu* is seen for *Pax6*.*2* in a number of gnathostome species such as lizard, *Xenopus*, zebrafish and elephant shark, but only in the latter is the *Fbxo47* gene found between *Pax6*.*2* and *Calu*. None of the immediate flanking genes in the sea lamprey *Pax6γ* locus (Fig. 1b) are conserved in any of the gnathostome *Pax6* loci.

### Expression pattern of lamprey *Pax6* genes

*Pax6* genes exhibit a strictly defined expression pattern^9,17,31^, with major sites of expression seen in specific areas of the developing eye and central nervous system (CNS). To obtain some initial insight into the expression patterns of the lamprey *Pax6* genes and to assess if the three genes would be distinguished by differences in their levels and tissue-specificity of expression we performed qRT-PCR analysis in a number of tissues. Consistent with other vertebrates, strong expression of all three lamprey genes was found in the eye, and at much lower levels also in brain tissue (Fig. 2b). In addition, expression of lamprey *Pax6α* and *Pax6β* was observed in the ovary, whereas *Pax6β* and *Pax6γ* expression was seen in a number of other tissues: kidney, intestine and ovary for *Pax6β*; and skin for *Pax6γ* (Fig. 2b). Previous *in situ* hybridization studies had shown that Japanese lamprey *Pax6β* is expressed in the eye, the nasohypophysial plate, the oral ectoderm and the fore- and hindbrain of embryos^25,26^.

We had previously generated RNA-seq data from the pancreas of juvenile (8 to 11 cm long) brook lamprey (*Lampetra planeri*) (GenBank accession number PRJNA369595)^32^. The pancreas in lampreys exists as a collection of islet-like cells known as the islet organ. We demonstrated expression of *Pdx*, insulin, glucagon, *Slc2a2*, *Foxa2*, *Hnf1a*, *Neurod1* and *Prkacb* in these cells, key factors involved in pancreas development and insulin secretion in gnathostomes. A TBLASTN search of the assembled transcripts using our newly identified Japanese lamprey *Pax6* protein sequences revealed two transcripts for *Pax6α* with TPM values of 0.08 and 0.22, respectively (see Supplementary Information) but not for *Pax6β* and *Pax6γ*. This indicates that *Pax6α* is specifically expressed in the lamprey pancreas and suggests that *Pax6*α was co-opted into the pancreas developmental network early during vertebrate evolution.

### Phylogenetic analysis

**Figure 3:**
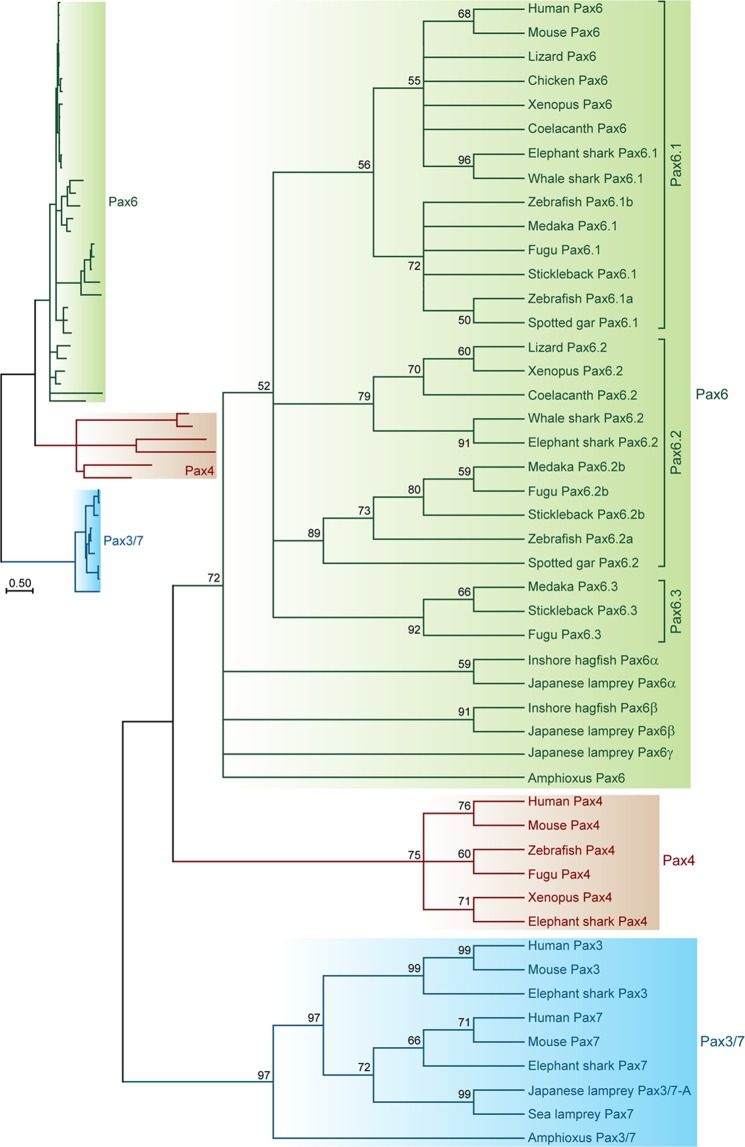
Maximum Likelihood tree of vertebrate *Pax6* genes. Phylogenetic analysis of the lamprey *Pax6* genes with *Pax6*, *Pax4*, *Pax3* and *Pax7* genes from several other chordate species. The Pax3/7 clade was specified as the outgroup. The phylogram is shown on the left and its cladogram on the right. The tree highlights clustering of the three lamprey genes with *Pax6* genes from other chordates thereby indicating that they are indeed lamprey *Pax6* genes.

To gain better insight into the relationship between the three lamprey *Pax6* genes and the members of the *Pax6*, *Pax4* and *Pax3/7* gene families from other chordates, we performed phylogenetic analysis using the Maximum Likelihood method. The ML tree showed that the three lamprey genes cluster with *Pax6* genes from other chordates distinct from the *Pax4* clade indicating that they are indeed *Pax6* genes (Fig. 3). However, the three lamprey *Pax6* genes clustered outside of the gnathostome *Pax6* clade (Fig. 3), a pattern previously observed for other lamprey gene families such as *KCNA*, *Hox*, *Runx*, and *p53*^20,33–35^, due to the exceptionally high GC-content in their coding regions that is peculiar to lampreys. The exclusive clustering of the lamprey genes outside the gnathostome clade rendered the analysis uninformative in terms of orthology assignment. We therefore named the three lamprey genes as *Pax6α*, *Pax6β* and *Pax6γ* (Fig. 1a).

### Conserved non-coding elements (CNEs) in the lamprey *Pax6* loci

**Figure 4:**
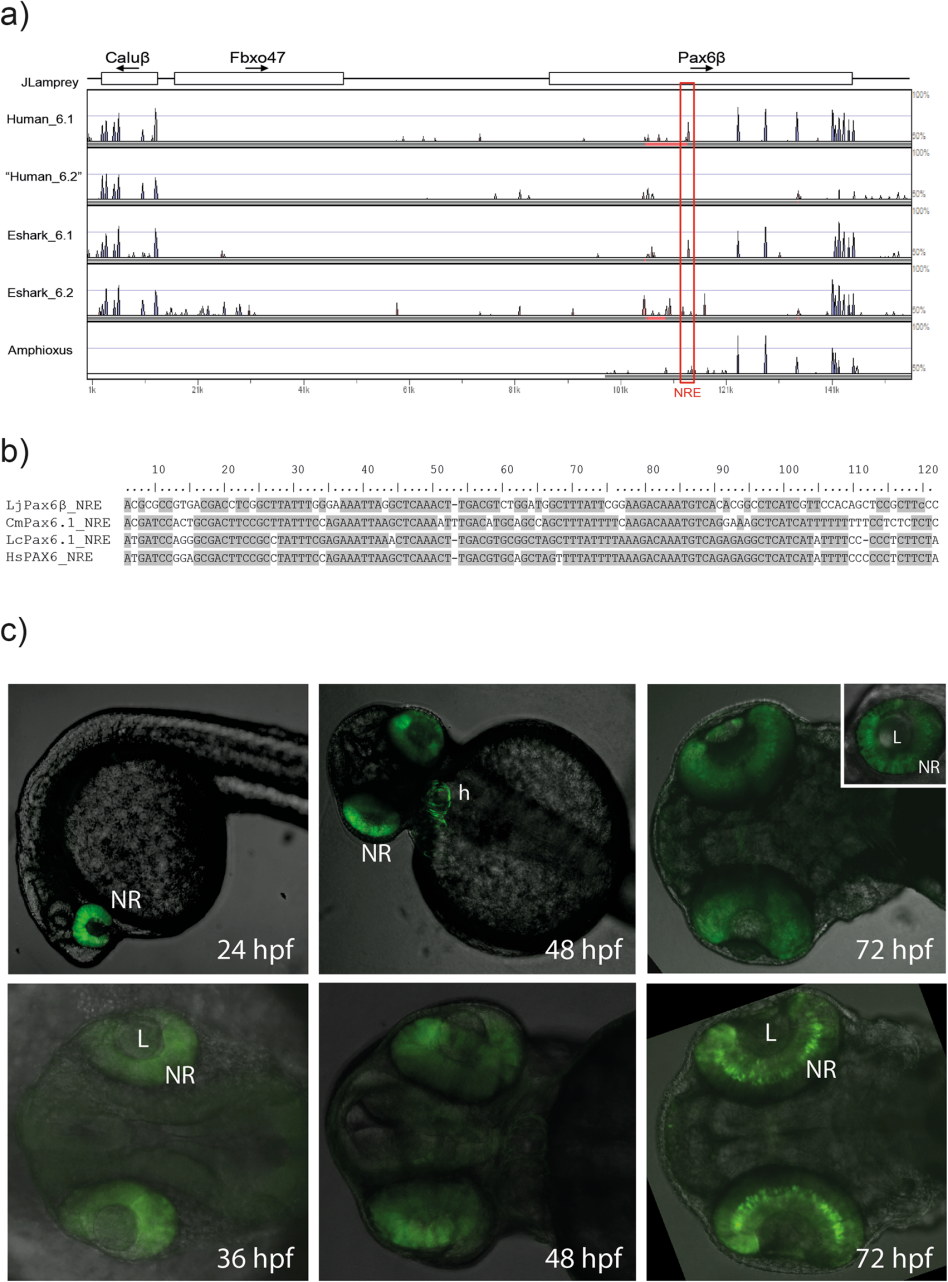
An ancient vertebrate conserved non-coding element is present in the lamprey *Pax6β* locus. (**a**) VISTA plot of the SLAGAN alignment of the *LjPax6β* locus, against the two *Pax6* loci from elephant shark and human, as well as the amphioxus locus. Note that there is no *PAX6* gene in the virtual ‘human_6.2’ locus. Sequence similarity outside of exons was seen for only a single element, homologous to the *PAX6* neuroretina enhancer (NRE). (**b**) Conservation at the core sequence of the NRE element. (**c**) Transgenic zebrafish assay of the *LjPax6β* putative NRE element. The Lj_NRE element was cloned in front of a minimal promoter-eGFP cassette and used to generate stable transgenic fish. Embryos at 24, 36, 48 and 72 hours post fertilisation (hpf) show highly specific and consistent GFP signal in the neuroretina of the developing eye. Ectopic expression in the heart due to site of integration effect was seen in one of the transgenic lines. Embryos were imaged from the lateral side (24 hpf and inset 72 hpf showing a close-up of the eye) or ventral side (36 hpf, 48 hpf and 72 hpf) with the fluorescent confocal signal overlaid on a brightfield view. L, lens; NR, neuroretina; h, heart.

Finally, we performed sequence alignments for each of the lamprey *Pax6* genomic scaffolds with *Pax6* loci from other species using SLAGAN^36^ to identify putative CREs. An mVISTA visualisation of the alignment of the lamprey *Pax6β* locus with human and elephant shark *Pax6* loci is shown in Fig. 4a. Only one distinct region of sequence similarity outside the exons was identified, located inside the lamprey *Pax6β* gene. On closer inspection, this element appeared to correspond to the neuroretina enhancer (NRE), a well-known *Pax6* enhancer which is located in intron 4 of the human *PAX6* gene^31,37^. The putative Lj_NRE shows 76% identity to the human NRE over an 88 bp core sequence, with 77% to coelacanth and 70% to the elephant shark *Pax6*.*1* NRE core (Fig. 4b). No significant sequence similarity outside the exonic regions was found for the other lamprey *Pax6* loci, nor could we detect any CNEs in alignments with the amphioxus *Pax6* locus (Fig. 4a). To examine the potential function of this lamprey *Pax6β* CNE, an 878 bp fragment covering the 88 bp core region plus flanking sequences (Fig. S2) was PCR amplified from the *Pax6β* locus and inserted into a fluorescence reporter construct for the production of stable transgenic zebrafish. From among many primary transgenic embryos, four independent transgenic lines were established. All expressing lines showed strong and specific GFP fluorescence in the retinae of transgenic fish at 24, 36, 48 and 72 hpf (Fig. 4c), with additional, variable ectopic expression seen in some individual lines due to site of integration of the transgene (Table S1). Fluorescence signal became primarily located to the inner nuclear layer (INL) in 72 hpf embryos (Figs. 4c; and S3). The highly specific retinal expression of the Lj_NRE element is very similar to the expression driven by NRE elements from mouse or elephant shark in zebrafish transgenics^13^, thus supporting a very ancient role for the NRE element as a retinal enhancer in the ancestral *Pax6* locus.

## Discussion

In a previous detailed analysis of *Pax6* gene loci in gnathostomes^13^, we found that unlike mammals, genomes of several vertebrate species possess multiple *Pax6* genes that are likely to have originated in the 2 R duplications. In the present study we have extended this analysis to the Japanese lamprey, representing the sister group of gnathostomes. We find that the lamprey genome contains three *Pax6* genes encoded by three separate genomic loci. The presence of more than two genes in its genome is consistent with a post-2R divergence of lampreys from gnathostomes. However, it has been proposed that an independent third WGD may have occurred in the lamprey lineage^20^. More recent studies have suggested that the two lineages shared only one WGD followed by a set of segmental duplications in the lamprey lineage^21,22^. This scenario is supported by analysis of synteny, which suggests similarity between the lamprey *Pax6α* and gnathostome *Pax6*.*1* loci, with the *Rcn1* homolog *Caluα* and *Elp4* flanking lamprey *Pax6α*/*Pax6*.*1*. The presence of *Fbxo47* between *Caluβ* and lamprey *Pax6β* is reminiscent of the gene content of the elephant shark *Pax6*.*2* locus.

All three lamprey *Pax6* genes identified in this study contain the paired box. The *Pax6*.*2* gene found in elephant shark and some other species lacks the paired domain-coding exons^13^. If lamprey *Pax6β* is indeed the ortholog of *Pax6*.*2*, as suggested by synteny, loss of the paired domain most likely occurred in the gnathostome lineage after the split between the cyclostome and gnathostome lineages, but before the divergence of the cartilaginous fish and bony vertebrate lineages.

In gnathostomes, *Pax6* is crucial for proper development and subsequent functioning of the eye, the central nervous system (CNS), the olfactory system and the pancreas^1–4^, and this is reflected in its tightly controlled expression in those tissues^3,17,31^. We found strong expression of all three genes in the eye, confirming the ancient role of *Pax6* in ocular development and function. Similarly its well-known importance in the brain is underscored by strong expression of the *LjPax6β* and *LjPax6γ* genes in the lamprey brain, with a lower level also seen in the *LjPax6α* expression pattern. Examination of RNA-seq data from the pancreas of a juvenile brook lamprey (*Lampetra planeri*) showed expression of *Pax6α*, but not *Pax6β* or *Pax6γ*, indicating a specific function for *Pax6*α in the lamprey pancreas. In jawed vertebrates, *Pax6* is crucial for proper development of the pancreas as well as the functional maintenance of the hormone-producing islet cells. In evolution, the pancreas is a novel endocrine organ that has come into existence with the emergence of the vertebrate lineage. Whereas in tetrapods the pancreas is a distinct organ that combines cells carrying out exocrine and endocrine roles, in cyclostomes the pancreas exists as a conglomeration of diffuse islet nodules associated with the gut area, similar to sharks and most bony fish^24,32,38^. We have previously shown that a number of key genes known to be crucial for pancreas development in gnathostomes, such as the transcription factors *Pdx1*, *Hnf1a*, *NeuroD1*, as well as *insulin* and *glucagon*, are expressed in the lamprey pancreas^32^. Our observation of *Pax6α* expression in the lamprey pancreas indicates that *Pax6* was also likely recruited early on into the gene network enabling the formation and development of this organ in the common ancestor of vertebrates.

In addition to the brain and eye, well-known vertebrate *Pax6* expression sites, expression of the lamprey *LjPax6β* and *LjPax6γ* genes was detected at low level in a number of other tissues. It remains to be investigated what role the genes play in these tissues. Interestingly, *LjPax6β* shows expression in the kidney. Kidney expression was also observed for elephant shark *Pax6*.*2*^13^. In mammals, *Pax6* expression is not observed in the kidney, but it is the main site of expression of the nearby Wilms tumour 1 (*Wt1*) gene^39^. We find no evidence for the presence of a *Wt1* homolog on our Japanese lamprey *Pax6* contigs (scaffolds 194 (1.1 Mb) and 23 (4.6 Mb)). A *Wt1* ortholog is present in the sea lamprey, but is located more than 7 Mb away from *Pax6β* beyond several intervening genes. Our longest contig, around the *Pax6α* gene, contains closely spaced homologs of *Eif3m* and *Calu* (a reticulocalbin (*Rcn*) family gene). In gnathostomes, including the elephant shark *Pax6*.*1* locus, where no *Pax6* kidney expression is seen, *Wt1* is situated between *Eif3m* and *Rcn1* (Fig. 1b), suggesting that it either got translocated to this position after the cyclostome divergence, or was lost independently in the lamprey. It is tempting to speculate that non-coding elements enabling kidney expression were present in the wider locus of the ancestral *Pax6* gene, which became functionally separated from the *Pax6* promoters early in the evolution of the *Pax6*.*1* loci (including the human *PAX6* locus), but were captured by a newly inserted *Wt1* gene.

The *Pax6* genomic region has long been a paradigm locus for understanding the principles of long range gene regulation and the evolution of *cis*-regulatory landscapes. We have previously studied the conservation of non-coding elements in a wide range of vertebrate *Pax6* loci, representing evolutionarily diverged lineages of increasing age^13,17,18^. Sequence comparisons of mammalian *Pax6* with the *Pax6* loci of the elephant shark revealed the presence of a large complement of CNEs between the species^18^. Many of these ancient gnathostome CNEs (agCNEs) have been shown to act as *cis*-regulatory elements, indicating that the *Pax6 cis*-regulatory landscape was laid down early in vertebrate evolution. In contrast, comparisons with the tunicate (*Ciona intestinalis*) or amphioxus *Pax6* loci did not yield any recognisable CNEs^13^. We had therefore anticipated that CNE analysis of the lamprey *Pax6* loci could reveal new insights into the hypothesis that the rapid emergence of the multitude of regulatory elements was triggered by the 2 R events^40^. VISTA analysis in our study uncovered only a single CNE peak mapping to the core sequence of the well characterised neuroretina enhancer (NRE) located in intron 4 of mammalian *Pax6*^31,37,41^. We show that conservation of this CNE extends to its function, as reporter expression driven by the lamprey *Pax6β* NRE element in transgenic zebrafish is very similar to the retinal expression driven by the orthologous elements from mouse or elephant shark^13^. This conservation of sequence and function of the NRE suggests that the element was already present and functional in the ancestral vertebrate locus. Apart from the NRE element, we did not detect any other CNEs in the three lamprey *Pax6* loci. Thus, compared to the large number of CNEs in elephant shark^18^, there appears to be a striking dearth of vertebrate CNEs around the lamprey *Pax6* genes (Fig. S4). It is possible that putative ancestral elements have been lost or diverged beyond recognition in the lamprey genome, but a more likely scenario suggests that the large numbers of CNEs were invented in the gnathostome lineage after the split between cyclostome and gnathostome lineages^40^. Undoubtedly, *cis*-regulatory elements also exist around the lamprey genes and comparisons between different lamprey species or with the hagfish genome may reveal such cyclostome-specific CNEs, even though the Japanese and sea lamprey are too closely related to be helpful in this respect. Similar to the lack of CNEs between gnathostome *Pax6* loci and those from *Ciona* or *Branchiostoma* (amphioxus) we detected no CNEs in sequence alignments between lamprey and amphioxus (Fig. 4a). In keeping with this, only a handful of CNEs were found when comparing 50 key developmental loci between cephalochordates and vertebrates^42^, even though many conserved sequences were identified when comparing the genomes of two cephalochordates, *Asymmetron lucayanum* and *Branchiostoma floridae*.

While functional genomics of the Mediterranean amphioxus (*Branchiostoma lanceolatum*) revealed many regions of open chromatin, hinting at the existence of a significant number of putative cis-regulatory elements in its genome, this number of potential cis-elements was nevertheless much lower than that typically found in gnathostomes, in particular when comparing regions around genes with highly restricted expression patterns^43^. Future detailed functional analysis of the genomic loci of pleiotropic developmental regulatory genes such as *Pax6* in lamprey and hagfish may provide insight into the extent of their cis-regulatory complexity. In gnathostomes *Pax6* is surrounded by a large array of enhancer elements, many of which drive similar or overlapping expression patterns^18^. Functional redundancy between cis-elements was demonstrated for two *Pax6* lens enhancers by investigation of the consequences of their separate and combined deletions in the mouse^10,44,45^. Similarly, in addition to the NRE, multiple cis-elements with overlapping enhancer activity in the developing retina have been found in the mammalian *Pax6* locus^17,46^. It remains to be investigated how their deletions in various combinations may affect retinal development, and to what extent these may recapitulate the dramatic impact on eye development seen with the conditional ablation of *Pax6* itself from retinal tissues at various stages of development^12^. Conditional *Pax6* deletion using a Cre transgene driven by the mouse NRE enhancer (αCre) led to exclusive amacrine cell formation in the central retina and premature activation of photoreceptor differentiation in the peripheral retina^11,47^. Ablation at an earlier stage in development using a different Cre driver caused general failure of retinal progenitor cells to proliferate properly, while retinal cells failed to follow a correct differentiation pathway upon post-natal removal of *Pax6*^12,48^. Formation of correct post-natal cellular circuitry in the retina was shown to be dependent on antagonistic activity on the NRE between *Pax6* itself and a complex of LIM domain containing proteins binding to the conserved core of the element^49^. Overexpression of *Pax6* also disrupts aspects of retinal development in a stage-dependent manner^48^, underlining the critical importance of spatio-temporally precise control of *Pax6* dosage. It is conceivable that in lampreys, where a relatively smaller number of cis-regulatory elements is presumed to exist around the individual *Pax6* genes (as well as other developmental control genes), precision and robustness in the regulatory control circuitry is dependent on the presence of multiple separate *Pax6* genes.

In summary, our work reveals that the lamprey genome contains three *Pax6* genes, all possessing a paired domain but lacking an alternatively spliced exon 5a. One of the lamprey *Pax6* genes (*Pax6α*) is specifically expressed in the pancreas, an organ characteristic of vertebrates. We identified only a single ancient CNE, in the lamprey *Pax6β* locus, and demonstrate its functional conservation as a neuroretina enhancer in a zebrafish reporter assay. This element therefore represents the oldest recognisable vertebrate *Pax6 cis*-regulatory element, displaying functional conservation over 500 million years of evolution.

## Materials and Methods

### Identification of *Pax*6 genes in the Japanese lamprey genome

The genome of the Japanese lamprey, having a relatively small genome size of 1.6 Gb, was recently sequenced by our group^20^. We searched for *Pax6* genes in this assembly (GenBank accession APJL00000000, LetJap1.0) using an available *Pax6* protein sequence from Japanese lamprey (GenBank accession BAB62531.1^25^), as well as those from human and elephant shark. Our TBLASTN searches picked up several *Pax* gene-containing scaffolds of which scaffold 23 (4.6 Mb) and scaffold 194 (1.09 Mb) were positive for *Pax6*. In addition, we identified fragments of another *Pax6* gene distributed across multiple scaffolds. We used the identified *Pax6*-containing scaffolds to design probes for screening Japanese lamprey BAC libraries. For the third *Pax6* gene, we were unable to identify any BAC clones and therefore we performed RT-PCR and RACE (Rapid Amplification of cDNA Ends) using cDNA from eye to generate full-length coding sequence and to orient scaffolds belonging to the same gene, and closed gaps using genomic PCR to obtain the complete sequence of this locus.

### Identification and sequencing of BACs

Three different Japanese lamprey BAC libraries (IMCB_Testis1: *Eco*RI, 92,160 clones, average insert size 100 kb; IMCB_Testis2: *Hin*dIII, 165,888 clones, average insert size 115 kb; and IMCB_Blood1: *Hin*dIII, 119,808 clones, average insert size 115 kb) were used to identify *Pax6*-containing BAC clones. The BAC libraries were screened using probes designed from the identified scaffolds and standard radioactive probing methods. Selected positive BACs were sequenced completely using the standard method of shotgun Sanger sequencing and gap filling by PCR or primer walking. Sequencing was done using the BigDye Terminator Cycle Sequencing Kit (Applied Biosystems, USA) on an ABI 3730xl capillary sequencer (Applied Biosystems, USA). Chromatograms were processed and assembled using Phred-Phrap^50^ and Consed^51^. Sequences for the three Japanese lamprey *Pax6* loci generated in this study have been submitted to GenBank with accession numbers MH778922-MH778924.

### Phylogenetic analysis

Phylogenetic analysis was carried out using *Pax6* genes from Japanese lamprey along with orthologues from representative tetrapods (human, mouse, chicken, Anole lizard, *Xenopus*), coelacanth, teleosts (fugu, medaka, stickleback, zebrafish), spotted gar, cartilaginous fishes (elephant shark and whale shark), inshore hagfish and the cephalochordate (amphioxus). In addition, we included Pax4 and Pax3/7 sequences from representative chordate species. Multiple alignments were generated using MAFFT version 7 web server (https://mafft.cbrc.jp/alignment/server/) using the L-INS-i strategy. Alignments were inspected manually using BioEdit sequence alignment editor^52^. A Maximum Likelihood (ML) tree was generated for this alignment using TREE-PUZZLE version 5.3.rc16^53^. We used ‘exact (slow)’ parameter estimation using the ‘quartet sampling plus NJ tree’ option, 10,000 puzzling steps, eight gamma rate categories and a JTT + G + F substitution model as deduced by ModelGenerator version 0.85 for the ML analysis. Amino acid frequencies and the gamma distribution parameter alpha were set to be determined from the dataset. The Pax3/7 clade was specified as the outgroup.

### Real-time qRT-PCR

Total RNA of adult Japanese lamprey (from a routine catch from commercial fishermen on the Ishikari River near Ebetsu in Hokkaido, Japan) was extracted from nine tissues (brain, eye, gills, heart, intestine, kidney, ovary, skin and testis) using the Trizol reagent (Life Technologies, Carlsbad, California) according to the manufacturer’s protocol. One µg of total RNA was reverse transcribed into 5′RACE-ready single strand cDNA using the SMART RACE cDNA Amplification kit (Clontech, Palo Alto, CA) and used as template for qRT-PCR using the SYBR Select Master Mix (Life Technologies). qRT-PCR primer sequences are listed in Table S2. The qRT-PCR was performed using the ViiA 7 Real-Time PCR System (Applied Biosystems, Foster City, CA) and SYBR Select Master Mix (Life Technologies) with the following cycling conditions: 50 °C for 2 minutes, 95 °C for 3 minutes, followed by 40 cycles of 95 °C for 3 seconds and 65 °C for 30 seconds. Quantification of gene expression levels was performed using the comparative CT method^54^. We used three technical replicates per tissue for each of the three *Pax6* genes and determined their average expression level (±Standard Error). Expression levels of the *Pax6* genes were normalized using the β*-actin* gene as the reference. The relative expression level of each *Pax6* gene between different tissues was estimated using the tissue with the lowest level of expression among the tissues analysed as reference tissue.

### Identification and analysis of CNEs

*Pax6* loci from Japanese lamprey, amphioxus, elephant shark and human were used for CNE prediction. The amphioxus (*Branchiostoma floridae*) sequence was extracted from the JGI Genome Portal (https://genome.jgi.doe.gov/), using assembly version Brafl1 with *Pax6* present on scaffold_23. Repetitive sequences were masked using the CENSOR web server^55^. Multiple alignments of the repeat-masked sequences were generated using the global alignment program SLAGAN^36^ with the Japanese lamprey sequence as the reference. CNEs were predicted using a cut-off of ≥65% identity across >50 bp windows and visualized using VISTA^56^. In addition, sequences of known CNEs between elephant shark and human were searched against the Japanese lamprey *Pax6* loci using BLASTN.

### Cloning of the lamprey CNE for the zebrafish enhancer assay

An 878 bp fragment containing the CNE from the Japanese lamprey *Pax6β* locus plus flanking sequence was cloned by PCR amplification using Phusion high fidelity polymerase (NEB). attB4 and attB1r sequences (underlined in the primers below) were attached to the PCR primers for use with the Gateway recombination cloning system (Invitrogen). The amplified fragment was inserted into the Gateway pP4P1r entry vector using BP clonase and the sequence was verified using M13 forward and reverse primers. Primer sequences used for amplification of the lamprey CNE are:

Lamp6β_NRE_FP-B4:

5′-AACGGGGACAACTTTGTATAGAAAAGTTGGGAGATCGTGATGGAGGTGT-3′ and Lamp6β_NRE_RP-B1r:

5′-AACGGGGACTGCTTTTTTGTACAAACTTGACCCCACGTGTACCGTCTAA-3′ Next, the lamprey CNE-containing pP4P1r entry construct was mixed with a pDONR221 construct containing a gata2 minimal promoter-eGFP-polyA cassette, and recombined using LR Clonase into a destination vector with a Gateway R4-R2 cassette flanked by Tol2 recombination sites to produce the Lj*Pax6*β-CNE-gata2-eGFP reporter construct. The minimal gata2 promoter-eGFP reporter cassette has been used to report on the tissue-specific expression patterns driven by a wide variety of linked enhancers and does not produce reporter expression without the presence of linked enhancer elements^57^.

### Generation of transgenic zebrafish

Maintenance of zebrafish and the generation of transgenic fish were done according to previously described procedures^57^. Lj*Pax6*β-CNE-gata2-eGFP reporter plasmid DNA was isolated using a Qiagen miniprep kit and further cleaned via a Qiagen PCR purification column. Tol2 transposase RNA was synthesized with the SP6 mMessage mMachine kit (Ambion) from a NotI-linearized pCS2-TP plasmid^58^. An injection mix containing 25 ng/μl each of the reporter plasmid DNA and transposase RNA was micro-injected into the cytoplasm of ~200 embryos at the 1- to 2-cell stage. Embryos showing mosaic fluorescence at 1–5 days post-fertilization (dpf) were raised to adulthood and used to establish lines.

Imaging of transgenic embryos was performed as previously described^57^. Embryos, treated with 0.003% PTU (1-phenyl2-thio-urea) from 24 hpf to repress pigmentation, were anaesthetised with tricaine and mounted in 1% low-melting agarose for imaging on a Nikon A1R confocal microscope.

### Ethics statement

The zebrafish experiments were approved by the University of Edinburgh Ethical Committee and performed under UK Home Office license number PIL PA3527EC3; PPL IFC719EAD. Extraction of DNA and RNA from lamprey tissues was approved by the Institutional Animal Care and Use Committee of the Biological Resource Centre, Agency for Science, Technology and Research (A*STAR), Singapore.

## Supplementary information

Supplementary Information

## Data Availability

Sequences for the three Japanese lamprey *Pax6* loci generated in this study have been submitted to GenBank with accession numbers MH778922-MH778924.
