# Supplementary material for: Lampreys, the jawless vertebrates, contain three Pax6 genes with distinct expression in eye, brain and pancreas
Source: Sci Rep. 2019 Dec 20;9:19559. doi: 10.1038/s41598-019-56085-8 (PMC6925180; doi:10.1038/s41598-019-56085-8)
Supplement: Supplementary file 1 — Supplementary Information [file 41598_2019_56085_MOESM1_ESM.pdf]

Supplementary Material

Lampreys, the jawless vertebrates, contain three *Pax6* genes with distinct expression in eye, brain and pancreas

Vydianathan Ravi[1], Shipra Bhatia[2], Prashant Shingate[1], Boon-Hui Tay[1], Byrappa Venkatesh[1,3*], Dirk A. Kleinjan[2,4*]

Table S1: eGFP expression in F1 zebrafish embryos in four independent stable reporter transgenic lines.

**Cis Regulatory Element (CRE)**	**Reporter cassette**	**Total number of stable transgenic lines analysed**	**Sites of reporter expression (**in number of lines out of the total**)**	**Tissue-specific activity observed in 100% of transgenic lines**
LjPax6β_NRE element	Minimal gata2 promoter-eGFP	4	Retina<br>(4/4; 100%)<br>Heart<br>(1/4; 25%)<br>Yolk (1/4; 25%)<br>Forebrain (1/4; 25%)	Retina

Table S2. Primer pairs used for the qRT-PCR of Japanese lamprey *Pax6* genes

Gene	Primer Name	Primer Sequences (5’→3’)
Pax6-α	LjPax6α_ex12_F2	5’-TACGATACGTACACGCCGCCACACA-3’
	LjPax6α_3utr_R1	5’-ACCGCCCATCGCAGGAGAGAGGC-3’
Pax6-β	LjPax6β_ex12_F2	5’-TACTCGTGCATGCTCCCCACGAG-3’
	LjPax6β_3utr_R2	5’-AGGCAGACAGGTAGGAGGAGCCA-3’
Pax6-γ	LjPax6γ_ex12_F2	5’-TCGAAGCGTACTCAAACAATCAGCC-3’
	LjPax6γ_3utr_R2	5’-TACTGGAGCCTGGACCAGTAGTGTGC-3’
β-Actin	LjBACTIN.F	5’-ATCGTGCGTGACATCAAGGAGAAG-3’
	LjBACTIN.R	5’-GCGTACAGGTCCTTGCGGATGTC-3’

Intron1

```
LjPax6α ATGAACAACAgtgag......gacagGCCACAGCGGC
         M  N  N                  S  H  S  G
LjPax6β TTTCGTCAAGgtgag......gccagGCCACAGCGGC
         F  R  Q                  G  H  S  G
LjPax6γ TATCAAAAAGgcaag......attagGTCACAGCGGG
         Y  Q  K                  G  H  S  G
HsaPax6 ATGCAGAACAgtaag......ctcagGTCACAGCGGA
         M  Q  N                  S  H  S  G
```

Intron2

```
LjPax6α ATCCTGCAGgtgcg......tgcagGTGTCCAAC
         I  L  Q                 V  S  N
LjPax6β ATCCTGCAGgcgag......cgcagGTGTCTAAC
         I  L  Q                 V  S  N
LjPax6γ CTACTGCAGgtgag......cccagGTCTCCAAC
         L  L  Q                 V  S  N
HsaPax6 ATTCTGCAGgtgat......tgcagGTGTCCAAC
         I  L  Q                 V  S  N
```

Intron3

```
LjPax6α ATCCCCAGCgtgag......cacagGTCTCCTCC
         I  P  S                 V  S  S
LjPax6β ATCCCCAGCgtgag......cccagGTGTCGTCC
         I  P  S                 V  S  S
LjPax6γ CTGCCCAGCgtgag......cgcagGTGTCGTCC
         L  P  S                 V  S  S
HsaPax6 ATACCAAGCgtaag......tgcagGTGTCATCA
         I  P  S                 V  S  S
```

Intron4

```
LjPax6α CTCTCAGCAGgtacg......gggagGCTCCGCGGGG
         L  S  A                  G  S  A  G
LjPax6β CAGGGGCCAGgtggg......cgcagACGGGTGCGCG
         Q  G  P                  D  G  C  A
LjPax6γ GGCGCCTTCGgtgag......gccagAGCTGGAGGGG
         G  A  F                  E  L  E  G
HsaPax6 CCTACGCAAGgtaaa......tccagATGGCTGCCAG
         P  T  Q                  D  G  C  Q
```

Intron5

```
LjPax6α CTCGAGAAAGgtggg......tgcagAGTTCGAGAGG
         L  E  K                  E  F  E  R
LjPax6β CTCGAGAAAGgtgag......cccagAGTTCGAGCGG
         L  E  K                  E  F  E  R
LjPax6γ CTGGAGAAAGgtgcg......tgtagAATTCGAACGG
         L  E  K                  E  F  E  R
HsaPax6 CTGGAGAAAGgtgat......ttcagAGTTTGAGAGA
         L  E  K                  E  F  E  R
```

Intron6

```
LjPax6α CGCATCCAGgtgag......cacagGTGTGGTTC
         R  I  Q                 V  W  F
LjPax6β CGCATCCAGgtgag......cccagGTGTGGTTC
         R  I  Q                 V  W  F
LjPax6γ AGGATCCAGgtaac......ctcagGTGTGGTTC
         R  I  Q                 V  W  F
HsaPax6 AGAATACAGgtacc......tccagGTATGGTTT
         R  I  Q                 V  W  F
```

...contd.

Intron7

```
LjPax6α GCGGCTCCCGgtacc......ccaagGCGCGATGCTG
          A  A  P                  G  A  M  L
LjPax6β ATGCACCCAGgtgag......cacagGTGCGATGCTG
          M  H  P                  G  A  M  L
LjPax6γ GCGCAACAAGgtcag......tgcagGTCAGATGCTG
          A  Q  Q                  G  Q  M  L
HsaPax6 ACCACACCGGgtaat......ctcagTTTCCTCCTTC
          T  T  P                  V  S  S  F
```

Intron8

```
LjPax6α CCCGTGCAGgtgag......cccagCCCGGCGTG
          P  V  Q                 P  G  V
LjPax6β CCCATGCAGgtgag......tccagGCGCCCATG
          P  M  Q                 A  P  M
LjPax6γ CCCAGCCAGgtgca......ctcagCCGACGTCC
          P  S  Q                 P  T  S
HsaPax6 CCTATGCAAgtaag......cacagCCCCCAGTC
          P  M  Q                 P  P  V
```

Intron9

```
LjPax6α TCCTCCACGGgtgag......tacagGGCTCATCTCC
          S  S  T                  G  L  I  S
LjPax6β GGGTCCACAGgtaaa......cccagGTCTCATCTCG
          G  S  T                  G  L  I  S
LjPax6γ GGATCCCCAGgtgag......tgtagGGATGCTGTCA
          G  S  P                  G  M  L  S
HsaPax6 ACTTCAACAGgtgag......tctagGACTCATTTCC
          T  S  T                  G  L  I  S
```

Figure S1. Comparison of exon-intron junctions of Japanese lamprey and human *Pax6* genes showing conservation of intron position and phase (also see Figure 2a). Intron numbering is based on the lamprey *Pax6α* gene (see Figure 2a). Upper case letters denote the exonic sequence whereas the lowercase letters represent the introns. The encoded amino acids are shown below their respective codons.

```
                                   10        20        30        40        50        60        70
                           ....|....|....|....|....|....|....|....|....|....|....|....|....|....|
Lamprey_Pax6β_NRE          -----------------------------------GGTTGGCCGTGGTGCTAGATGATG--ATGA-----
Eshark_6.1_NRE             ------------------GCTAGAACCTTCGCATCTGTGAACAGTTGTGAACTAAGTAGGACGCGCG--T
Coelacanth_Pax6_NRE        ---------------------------------------------------AGTTTT-AATAAAGCGC
Human_PAX6_intron_4_NRE    GCGCTGGTCCCGGCCGGAGCCCAGATCCCCCAAACGCCTGTCACTTCTCATCGAGTTGAGGCTCCGGCCC

                                   80        90       100       110       120       130       140
                           ....|....|....|....|....|....|....|....|....|....|....|....|....|....|
Lamprey_Pax6β_NRE          ---------TGATGGTGACGACGGTTTGTA----GGGAAGGAT-GGCGACAAGAGTTGTGGAGATgGAGG
Eshark_6.1_NRE             TCCCCCCCAATGTGGTGCCCCAGCCTCGCCCCCGGGGCAACCTTTGGAAACGAAGCTTTCTTTTTTTAAG
Coelacanth_Pax6_NRE        GCC--------------CCCGCAG-TTGTGCACGTATAAGCCTTTGGCTACC------TTAAAATGAAGC
Human_PAX6_intron_4_NRE    CCACCCGCGCCTTGGTGAGGGCCCCCCCTCAGCCCCAAGCCTTCGGCTACCC------TGAAAACGCGGC

                                  150       160       170       180       190       200       210
                           ....|....|....|....|....|....|....|....|....|....|....|....|....|....|
Lamprey_Pax6β_NRE          AGGAAGGGGAGGGGGGGTGAGAGAAGAGAGAAGAAGGGGGGGGGGGTGAAACGTCGCCGCCATCGCCACT
Eshark_6.1_NRE             GGGGTAATGAGCGGAGAGTGGAGCGAGC----GATAGGACTATGAGTGATACACAAATGCGGACAGAA--
Coelacanth_Pax6_NRE        TTCCCTTGAAGGGTAAATGAGTAGAATC----CAATTTAGTCTGAGTGATAACTAAATGCGGACAGAA--
Human_PAX6_intron_4_NRE    TCCCCTCGAAGGGGAAACAAGTGCAGTT----CAATCTCGTCTGAGTGATCTACAAATAAGGACGGAA--

                                  220       230       240       250       260       270       280
                           ....|....|....|....|....|....|....|....|....|....|....|....|....|....|
Lamprey_Pax6β_NRE          CGGCAGTCGCTCGCCCGGGCTGCAAAAAAAAATCCAcCCCCCCCCCCACCACCACCACCACCACAACTTG
Eshark_6.1_NRE             --AGAGACGTTTTATCATGCTACTATTTGTCGTG--------------------ACGATGCGATTTTCA-
Coelacanth_Pax6_NRE        --AGGGTCGTTTTATCATGCTACTATTTGTCGTG--------------------ACGATGCAATTTTCAA
Human_PAX6_intron_4_NRE    --AGGGTCGTTTTATCACGGTCCCGTTTGTCGTG--------------------ACGATGCAATTTCCCG

                                  290       300       310       320       330       340       350
                           ....|....|....|....|....|....|....|....|....|....|....|....|....|....|
Lamprey_Pax6β_NRE          AAGCAAACTTGTGACGCGATGTGACAAGGCTGGCAA---------TGCCGACTCTGCGCGCTGGAGCGTG
Eshark_6.1_NRE             AAGTAGAACACTGTCATAAAGTGACATGCCTGCCACAAGTGCTCTCACTGATCTTTTCAATTACCCTTCC
Coelacanth_Pax6_NRE        AAGCAGAGCACTGTCATAAAGTGACATGCCTGCCACAAGTGCTCCAACTGATCTTTTCAATTAGCCTTCC
Human_PAX6_intron_4_NRE    GAGCGGAGCACTGTCACAAAGTGACAAGGCTGCCACAAGCGCCCCGACTGATCTTTTCAATTAGCCTTCC

                                  360       370       380       390       400       410       420
                           ....|....|....|....|....|....|....|....|....|....|....|....|....|....|
Lamprey_Pax6β_NRE          CCTAACGCGCCGTGACGACCTCGGCTTATTTGGGAAAATTAGGCTCAAACT-TGACGTCTGGATGGCTTT
Eshark_6.1_NRE             GTGCACGATCCACTGCGACTTCCGCTTATTTCCAGAAATTAAGCTCAAAATTTGACATGCAGCCAGCTTT
Coelacanth_Pax6_NRE        ATGCATGATCCAGGGCGACTTCCGCCTATTTCGAGAAATTAAACTCAAACT-TGACGTGCGGCTAGCTTT
Human_PAX6_intron_4_NRE    ATGCATGATCCGGAGCGACTTCCGCCTATTTCCAGAAATTAAGCTCAAACT-TGACGTGCAGCTAGTTTT

                                  430       440       450       460       470       480       490
                           ....|....|....|....|....|....|....|....|....|....|....|....|....|....|
Lamprey_Pax6β_NRE          ATTCGGAAGACAAATGTCACACGGCCTCATCGTTCCACAGCTCCGCTTcCCCCCCCCCCCAACTTATAT
Eshark_6.1_NRE             ATTTTCAAGACAAATGTCAGGAAAGCTCATCATTTTTTTTTCCTCTCTCTCTCTCTCTCACTCTCTCT
Coelacanth_Pax6_NRE        ATTTTAAAGACAAATGTCAGAGAGGCTCATCATATTTTCC-CCCTCTTCTATATTTGGAGCTTATT----
Human_PAX6_intron_4_NRE    ATTTTAAAGACAAATGTCAGAGAGGCTCATCATATTTTCCCCCCTCTTCTATATTTGGAGCTTATT----

                                  500       510       520       530       540       550       560
                           ....|....|....|....|....|....|....|....|....|....|....|....|....|....|
Lamprey_Pax6β_NRE          CGGGTCG-------------------------------GGGAGGGAGCTTTGGAGTCGCACTCGGAGGA
Eshark_6.1_NRE             CTCCCCCCTTCTATATTTGGAGGCTTATTTATTGCTAAGTGGCTAGGCTTTGGGAGCCAATTTAGCAAGG
Coelacanth_Pax6_NRE        ----------------------------TATTGCTAAGGAGCCTAGAATCTTTGAGTCAATTTAGCAGGA
Human_PAX6_intron_4_NRE    ----------------------------TATTGCTAAGAAGCTCAGGCTCCTGGCGTCAATTTATCAGTA

                                  570       580       590       600       610       620       630
                           ....|....|....|....|....|....|....|....|....|....|....|....|....|....|
Lamprey_Pax6β_NRE          GTCTCGCGGACGAAGCCGCGGCTGGCGCGCGCCGCCGTTGATCGCGCGCGCGACAcCCCCCCCC
Eshark_6.1_NRE             CGCTGTTTGGAGAGGAGAACAG----------GTCTAGC------------TAGAAGCACAGCTTCTCCA
Coelacanth_Pax6_NRE        GGCTCCAAGGAGAGGAGAGCAG----------AGAAATAGAAAGAACACAGTTTCTCCAGGGAGGCTTCT
Human_PAX6_intron_4_NRE    GGCTCCAAGGAGAAGAGAGGAG----------AGGAGAGGAGAGCTGAACAGGGAGCCACGTCTTTTCCT

                                  640       650       660       670       680       690       700
                           ....|....|....|....|....|....|....|....|....|....|....|....|....|....|
Lamprey_Pax6β_NRE          CCCCCCAGTACCGTAGCAGCCTTCGCTTCCTTCCTGAGCCCTGAGCCAGATC--GGGCAGATCGCCAGTT
Eshark_6.1_NRE             GGGAGAAGGCGACAGGCACCCTGAGCCTCAGGTTCGGAGTCTCAATACGCCCTTCAACTGATGCC-----
Coelacanth_Pax6_NRE        GTAA-------------ACCTCAGCTTCAGGTTTGGGAACTGAAACTAGTGG-CTTCTGTTATCTTACC
Human_PAX6_intron_4_NRE    GGGAGGGCTGCTATCTAAGTCGGGGCTGCAGGTTGGAGATTTTTAAGGAAGTGGAAATTGGCAATTGGCT

                                  710       720       730       740       750       760       770
                           ....|....|....|....|....|....|....|....|....|....|....|....|....|....|
Lamprey_Pax6β_NRE          TTATTTTGTTCTTCTTCTTCGTAACGCCACCCGTCGCATTCCGC-------CTGCTGCCTGTCTCTCCCC
Eshark_6.1_NRE             TTTTTTTTTG---CTCTTAATAAGCCAA-AGAACAGATTGTGTTTTTTTAGTACATTGGGTGTTTGTCG
Coelacanth_Pax6_NRE        AATTGTAAA-------------------------------------------------------------
Human_PAX6_intron_4_NRE    TTGTGTGTCTGTGGTTTTTGGGGAGGGGG-ACTACAAA--------------------------------

                                  780       790       800
                           ....|....|....|....|....|....|
Lamprey_Pax6β_NRE          GCGTGAGtCCCGGGACGCTGGC--------
Eshark_6.1_NRE             TAATTGATGCATGCAGCCTTGCCATGCACA
Coelacanth_Pax6_NRE        ------------------------------
Human PAX6 intron 4 NRE    ------------------------------
```

Figure S2: Sequence alignment of the conserved sequence element from the Lamprey Pax6β locus with the neuroretina enhancer sequences from human, coelacanth and elephant shark. The most highly conserved core of the element is highlighted with a red box.

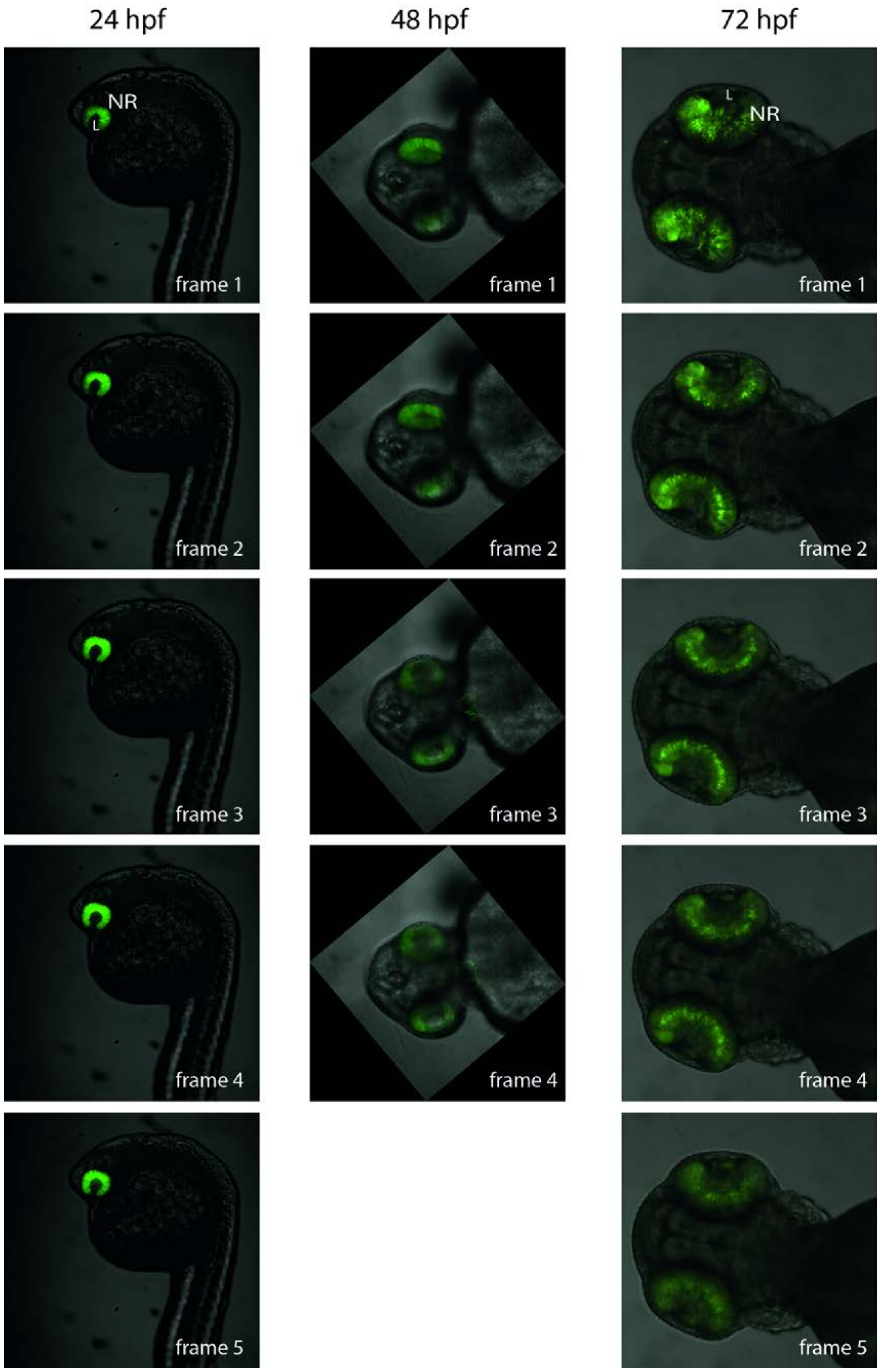

Figure S3: Optical sections of transgenic zebrafish embryos with the LjPax6β putative NRE element. A selection of ordered confocal z-stack images are shown for embryos at 24hfp, 48hfp and 72hpf.

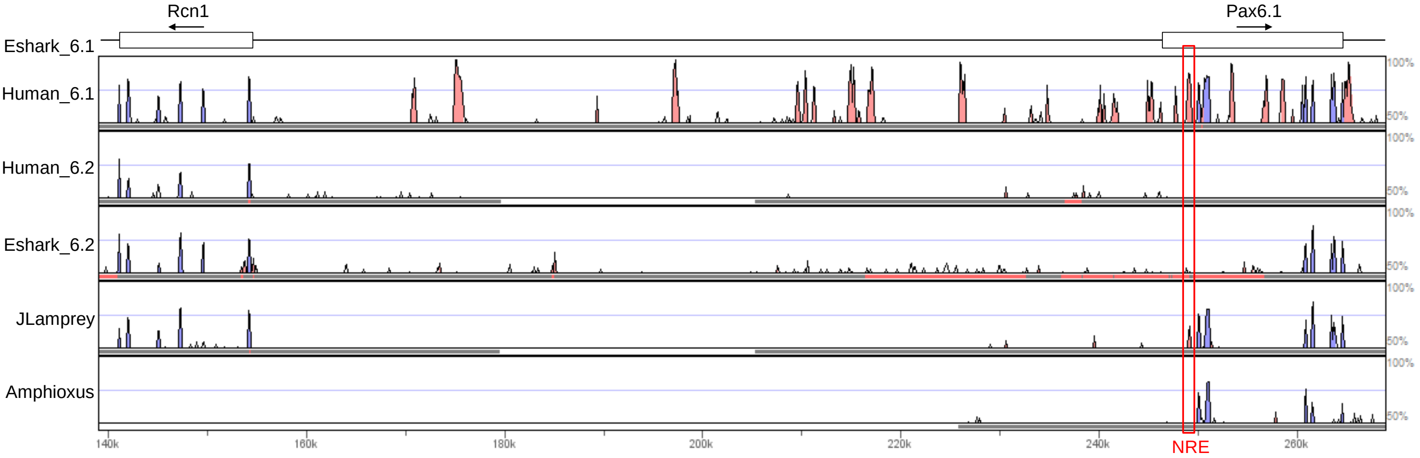

Figure S4: Appearance of a large number of conserved non-coding elements (CNEs) in gnathostomes after divergence from the lamprey. VISTA plot of the SLAGAN alignment of the elephant shark Pax6.1 upstream locus against Pax6 loci from human, elephant shark, the lamprey pax6β locus and the amphioxus pax6 locus. In contrast to the many CNEs conserved between the elephant shark and human Pax6.1 loci (pink shaded peaks; conserved coding sequences: blue shaded peaks), no CNEs are found in the other alignments apart from the lamprey Pax6β NRE (highlighted by red box). Note that there is no PAX6 gene in the virtual 'human_6.2' locus.

Pax6α transcripts in the RNA-seq transcript data of the brook lamprey (*Lampetra planeri*) pancreas.

>TRINITY_DN417217_c0_g1_i1 len=414 (Number of reads: 18; Transcripts per million (TPM) = 0.08)

CACAGCGGCGTGAACCAGCTGGGAGGAGTGTTCGTCAACGGGCGGCCGCTCCCCGACTCCATCCGC
CAGAAGATCGTCGAGTTAGCGCACAGCGGCGCGCGGCCCTGCGACATCTCCCGCATCCTGCAGGTG
TCCAACGGCTGCGTGAGCAAGATCCTGGGCCGCTACTACGAGACGGGCTCCATCCGCCCGCGCGCC
ATCGGCGGCAGCAAGCCGCGCGTGGCCACGCCCGAGGTGGTGGGGAAGATCGCGCAGTACAAGCGG
GAGTGCCCCTCCATCTTCGCGTGGGAGATCCGCGACCGCCTCCTCTCCGACGGGGTTTGCACCAGC
GACAACATCCCCAGCGTCTCCTCCATCAACCGGGTGCTGCGCAACCTGGCGAGCGAGAAGCAGGGG
ATGGGGGGCACCGACGGC

>protein

HSGVNQLGGVFVNGRPLPDSIRQKIVELAHSGARPCDISRILQVSNGCVSKILGRYYETGSIRPRA
IGGSKPRVATPEVVGKIAQYKRECPSIFAWEIRDRLLSDGVCTSDNIPSVSSINRVLRNLASEKQG
MGGTDG

>TRINITY_DN125441_c0_g1_i1 len=393 [TPM = 0.22]

AGCCACATCCCCATCAGCAGCAACTTCAGCGCGGGGGTCTACCAGCCCCTGGCGCAGCCCGCGGCT
CCCGGCGCGATGCTGGGCCGCAGCGACTCGATGCTCGGGGGCTCGTACGGGGCTCTACCCTCCATG
CCCAGCTTCAGCATGGCGGGGAACCTCCCCGTGCAGCCCGGCGTGGGCACGCAGACGGCCTCCTAC
TCGTGCATGCTGCCCTCCAACCCCGCCGTGTCGTCTTCGCGCGCCTACGACACGTACACGCCGCCA
CACATGCAGGCGGGCCACATGGGCACGGGGCCCTCCTCCACGGGGCTCATCTCCCCGGGGGTCTCG
GTTCCCGTGCAGGTGCCTGGTACTGACCCGGATCTCCCCCAATACTGGCCGCGTCTACAGTAG

>protein

SHIPISSNFSAGVYQPLAQPAAPGAMLGRSDSMLGGSYGALPSMPSFSMAGNLPVQPGVGTQTASY
SCMLPSNPAVSSSRAYDTYTPPHMQAGHMGTGPSSTGLISPGVSVPVQVPGTDPDLPQYWPRLQ

Full-length sequence of the Lj_Pax6β NRE fragment used to make reporter transgenic zebrafish

GGAGATCGTGATGGAGGTGTCCAAGACGCGACCGTGCCCGAGTCCGTGTCATCTGTGTGTGGGTGG
GTGTCGTGGGGGGGGGGGGGGTTTGGGAAGTGTGTCTGTATGGCGGGCGGGGTTGGCCGTGGTGCT
AGATGATGATGATGATGGTGACGACGGTTTGTAGGGAAGGATGGCGACAAGAGTTGTGGAGATGGA
GGAGGAAGGGGAGGGGGGGTGAGAGAAGAGAGAAGAAGGGGGGGGGGGTGAAACGTCGCCGCCATC
GCCACTCGGCAGTCGCTCGCCCGGGCTGCAAAAAAAAATCCACCCCCCCCCCCACCACCACCACCA
CCACAACTTGAAGCAAACTTGTGACGCGATGTGACAAGGCTGGCAATGCCGACTCTGCGCGCTGGA
GCGTGCCTAACGCGCCGTGACGACCTCGGCTTATTTGGGAAAATTAGGCTCAAACTTGACGTCTGG
ATGGCTTTATTCGGAAGACAAATGTCACACGGCCTCATCGTTCCACAGCTCCGCTTCCCCCCCCCC
CCCAACTTATATCGGGTCGGGGAGGGAGCTTTGGAGTCGCACTCGGAGGAGTCTCGCGGACGAAGC
CGCGGCTGGCGCGCGCGCCGCCGTTGATCGCGCGCGCGCGACACCCCCCCCCCCCCCCCCCAGTACC
GTAGCAGCCTTCGCTTCCTTCCTGAGCCCTGAGCCAGATCGGGCAGATCGCCAGTTTTATTTTGTT
CTTCTTCTTCGTAACGCCACCCGTCGCATTCCGCCTGCTGCCTGTCTCTCCCCGCGTGAGTCCCGG
GACGCTGGCGAGGGGACTGCCGCTGCGGGAGATCGGGCGGGGCAGACGGGCAGCCGGACGTGCTTG
TTAGACGGTACACGTGGGGT
